# Brca1 is expressed in human microglia and is dysregulated in human and animal model of ALS

**DOI:** 10.1186/s13024-015-0023-x

**Published:** 2015-08-01

**Authors:** Harun Najib Noristani, Jean Charles Sabourin, Yannick Nicolas Gerber, Marisa Teigell, Andreas Sommacal, Maria dM Vivanco, Markus Weber, Florence Evelyne Perrin

**Affiliations:** Institute for Neurosciences of Montpellier (INM), INSERM U1051, 80, rue Augustin Fliche, 34091 Montpellier, Cedex 5 France; “Integrative Biology of Neurodegeneration”, IKERBASQUE Basque Foundation for Science and Neuroscience Department, University of the Basque Country, Bilbao, Spain; Kantonspital St. Gallen. FachMuskelzentrum/ALS clinic, St. Gallen, Switzerland; CIC bioGUNE, Cell Biology & Stem Cells Unit, Technological Park of Bizkaia, Derio, Spain; Department “Biologie-Mécanismes du Vivant” Faculty of Science, University of Montpellier, Montpellier, France

**Keywords:** Microglia, Transcriptomics, hSOD1^G93A^ mice, ALS patients, Brca1

## Abstract

**Background:**

There is growing evidence that microglia are key players in the pathological process of amyotrophic lateral sclerosis (ALS). It is suggested that microglia have a dual role in motoneurone degeneration through the release of both neuroprotective and neurotoxic factors.

**Results:**

To identify candidate genes that may be involved in ALS pathology we have analysed at early symptomatic age (P90), the molecular signature of microglia from the lumbar region of the spinal cord of hSOD1^G93A^ mice, the most widely used animal model of ALS. We first identified unique hSOD1^G93A^ microglia transcriptomic profile that, in addition to more classical processes such as chemotaxis and immune response, pointed toward the potential involvement of the tumour suppressor gene breast cancer susceptibility gene 1 (Brca1). Secondly, comparison with our previous data on hSOD1^G93A^ motoneurone gene profile substantiated the putative contribution of Brca1 in ALS. Finally, we established that Brca1 protein is specifically expressed in human spinal microglia and is up-regulated in ALS patients.

**Conclusions:**

Overall, our data provide new insights into the pathogenic concept of a non-cell-autonomous disease and the involvement of microglia in ALS. Importantly, the identification of Brca1 as a novel microglial marker and as possible contributor in both human and animal model of ALS may represent a valid therapeutic target. Moreover, our data points toward novel research strategies such as investigating the role of oncogenic proteins in neurodegenerative diseases.

**Electronic supplementary material:**

The online version of this article (doi:10.1186/s13024-015-0023-x) contains supplementary material, which is available to authorized users.

## Background

Amyotrophic lateral sclerosis (ALS) is characterised by selective motoneurones degeneration in the spinal cord, brainstem and motor cortex leading to progressive muscle weakness, atrophy and paralysis. Approximately 90 % of ALS patients are sporadic whilst 10 % are familial cases with genetic mutations in SOD1 (Cu/Zn superoxide dismutase 1), FUS (fused in sarcoma), TARDBP (also known as TDP-43) and C9ORF72, among others [1]. Transgenic mice over-expressing the human mutated gene for SOD1 develop an adult-onset paralysis that closely recapitulates human ALS [2]. Recent studies have established that ALS is a complex multi-factorial disease that involves several cellular partners including glial cells [3].

Microglia, the resident immune cells of the central nervous system (CNS), when activated, release pro- and anti-inflammatory cytokines and chemokines that are generally associated with M1 and M2 phenotypes [4, 5]. Microglia have a dual role in ALS with an early protective effect on motoneurones but also a detrimental effect due to the secretion of neurotoxic factors [6]. It is hypothesised that progressive motoneurone death results from the combination of intrinsic motoneurones vulnerability and toxicity from neighbouring cells such as microglia [6]. In ALS patients and animal models, there is a clear microglia activation [3], in particular we have shown an early involvement of microglia in hSOD1^G93A^ mice [7]. Understanding the contribution of microglia to motoneurone degeneration is of high priority. One means of analysing the role of a cell population in a process network is to study gene expression alterations in this given population. In addition, an integrative comparison of the specific molecular signatures of several cellular partners is necessary to decipher the crosstalk between these cells. We have previously identified gene dysregulation in pure motoneurones from the lumbar spinal cord of hSOD1^G93A^ mice [8] and two other mouse models of motoneurone disease [9]. We revealed a unique motoneurone gene expression profile characterised by an absence of dysregulation of genes associated with cell death and a massive up-regulation of genes involved in cell growth [8].

Growing evidence points toward mitochondrial dysfunction and oxidative DNA damage in ALS [10]. Defence mechanisms, including SOD, counteract excessive accumulation of reactive oxygen species, however in ALS, cellular antioxidant defences are insufficient leading to damage of nucleic acids, proteins and lipids [11]. Inherited mutations in breast cancer susceptibility gene 1 (*Brca1*), a well-known tumour suppressor implicated in familial breast and ovarian cancers, is one of the best defined risk factor for development of breast and ovarian cancer. *Brca1* plays important roles in a broad spectrum of functions including transcription regulation, cell cycle checkpoint activation, apoptosis, chromosomal remodelling, ubiquitination and DNA repair [12]. The role of *Brca1* in each of these processes remains to be fully understood but it is hypothesized that it act as a scaffold for the formation of complexes with a wide range of proteins [13]. This ability of *Brca1* to interact with different proteins may underlie its involvement in a variety of cellular processes [13]. *Brca1* also exerts a protective role against oxidative stress via up-regulation of antioxidant genes and maintenance of the redox balance through up-regulating the expression of heat shock protein HSP27 [14, 15].

In breast cancer, *Brca1* cellular localisation as well as the significance of its altered localisation, is still a matter of debate. It had been recently shown that in normal breast, *Brca1* nuclear expression is strong and uniform in parenchymal cells whereas in malignant cells its expression is reduced if not absent from the nucleus and is, in some cases, observed in the cytoplasm [16]. Interestingly, altered expression of *Brca1* was associated with poor prognosis and shortened survival. In the adult rodent CNS, the presence of *Brca1* is detected only in neurons [17] whereas a high *Brca1* expression is observed in embryonic [17, 18] and adult neural stem cells and is involved in cell proliferation [18].

Here we identify putative *Brca1* involvement in ALS via hSOD1^G93A^ microglia gene profiling and comparisons to our previous transcriptomic findings in hSOD1^G93A^ motoneurones. We then demonstrated that *Brca1* is a novel marker of human microglia and is up-regulated in ALS patients.

## Results

### Transcriptomic analysis of FACS isolated microglia from control and hSOD1^G93A^ lumber spinal cord

**Fig. 1:**
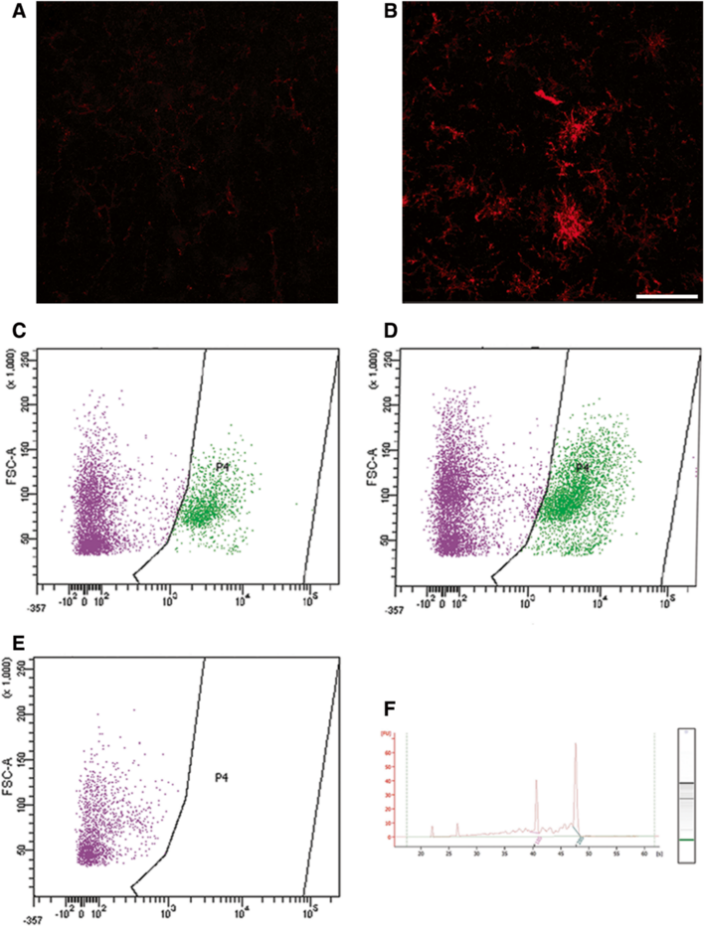
FACS analysis of CD11b^+^ microglia in control and hSOD1^G93A^ mice from the lumbar segment of the spinal cords at 90 days of age. Microglia were sorted by flow cytometry using the microglia marker CD11b. **a** Confocal images of CD11b expression in spinal cord microglia from control at 90 day of age and (**b**) from transgenic hSOD1^G93A^ mice at early symptomatic age. *Scale bars* (**a & b**): 50 μm. **c**–**d** Representative flow cytometry analysis dot plot displaying microglia profiles. **c** Control and (**d**) hSOD1^G93A^ spinal microglia at P90. In both (**c**) control and (**d**) hSOD1^G93A^ surrounded areas, designed as “*P4*”, correspond to the labelled cells. **e** Negative control (without CD11b labelling). The X-axis represents the intensity of fluorescence and the Y axis the size of the cells. **f** RNA quality isolated from FACSed microglia

We have previously described early microglial disturbances in hSOD1^G93A^ male mice reflected at P90 by a heterogeneous Iba1^+^ microglial distribution with higher density within the grey matter in hSOD1^G93A^ mice as compared to control [7, 19]. Since activated microglia/macrophages exhibit increased CD11b expression, we carried out CD11b immunostaining (Fig. 1a & b). CD11b-positive microglia displayed enlarged somata with short and thick processes that are typical of a reactive phenotype and were predominantly found in hSOD1^G93A^ mice (Fig. 1b). To further analyse transcriptomic modification specifically in microglia, we isolated microglia of hSOD1^G93A^ and control littermate males at early symptomatic age (P90) from the lumbar spinal cord (L1-L5) that corresponds to the onset of degeneration. Microglia were isolated by fluorescence-activated cell sorting (FACS) using CD11b (Fig. 1c–e). We observed a 1.65-fold increase in the total number of CD11b^+^ microglia in hSOD1^G93A^ versus controls (26 350; *n* = 15 in hSOD1^G93A^ and 15 900; *n* = 26 in control; Fig. 1c & d). RNA extracted from FACS purified microglia was of high quality (Fig. 1f) and microarrays analysis revealed 630 dysregulated genes (260 down-regulated and 370 up-regulated, Additional file 1: Table S1).

### Cross-talk between microglia and motoneurones

We had previously identified dysregulated genes in hSOD1^G93A^ motoneuronse during disease progression [8, 9]. To unravel potential molecular cross-talk between microglia and motoneurones, we performed a comparative analysis of gene dysregulation in both cell populations. Comparison of dysregulated genes at P90 between motoneurones (102 genes) and microglia (668 genes) revealed 19 common genes (Additional file 2: Figure S1A). Process network rankings were clearly different in the commonly dysregulated genes (in motoneurones and microglia) and uniquely dysregulated genes. Antigen presentation was classified first in the common group, whilst cytoskeleton and cytoplasmic microtubules genes were top ranked in motoneurones only set (Additional file 3: Table S2A). Similarly, cellular processes analysis ranked first immune response and antigen presentation in the common group whereas response to stress, regulation of immune response, system development and wounding response were the top 4 ranked processes in motoneurones only group (Additional file 3: Table S2B). Signalling and metabolic pathway analysis revealed immune response and cytoskeleton remodelling as first ranked in the commonly and motoneurone only dysregulated genes, respectively (Additional file 3: Table S2C).

We have previously shown that microglial reactivity precedes neuronal death in hSOD1^G93A^ mice [7]; to seek for potential modifications in microglia that could trigger motoneurone death, we compared dysregulated genes at P90 in microglia and P120 in motoneurones. Our previous microarrays analysis of microdissected motoneurones at the end stage of the disease (P120) showed no dysregulation of genes associated with cell death [8], this most likely reflects that dissected motoneurones were at an early demise stage. Indeed, we selected motoneurones that had an identifiable nucleus and a diameter of at least 25 μm, picking a sub-population of neurones that may resist degeneration. Three hundred twenty genes were uniquely dysregulated in hSOD1^G93A^ motoneurones; 603 uniquely dysregulated in hSOD1^G93A^ microglia; 65 genes were common (Additional file 2: Figure S1B). Clear differences were highlighted not only between the genes that were commonly and uniquely dysregulated but also in the ranking as compared to the previous analysis (microglia and motoneurones at P90, Additional file 4: Table S3). Particularly, inflammation and immune response were ranked top in motoneurones (Additional file 4: Table S3A). Interestingly, signalling and metabolic pathway analysis revealed the involvement of heme metabolism and DNA damage in both motoneurones and microglia (Additional file 4: Table S3C).

### Unique transcriptomic profiles of hSOD1^G93A^ microglia

**Fig. 2:**
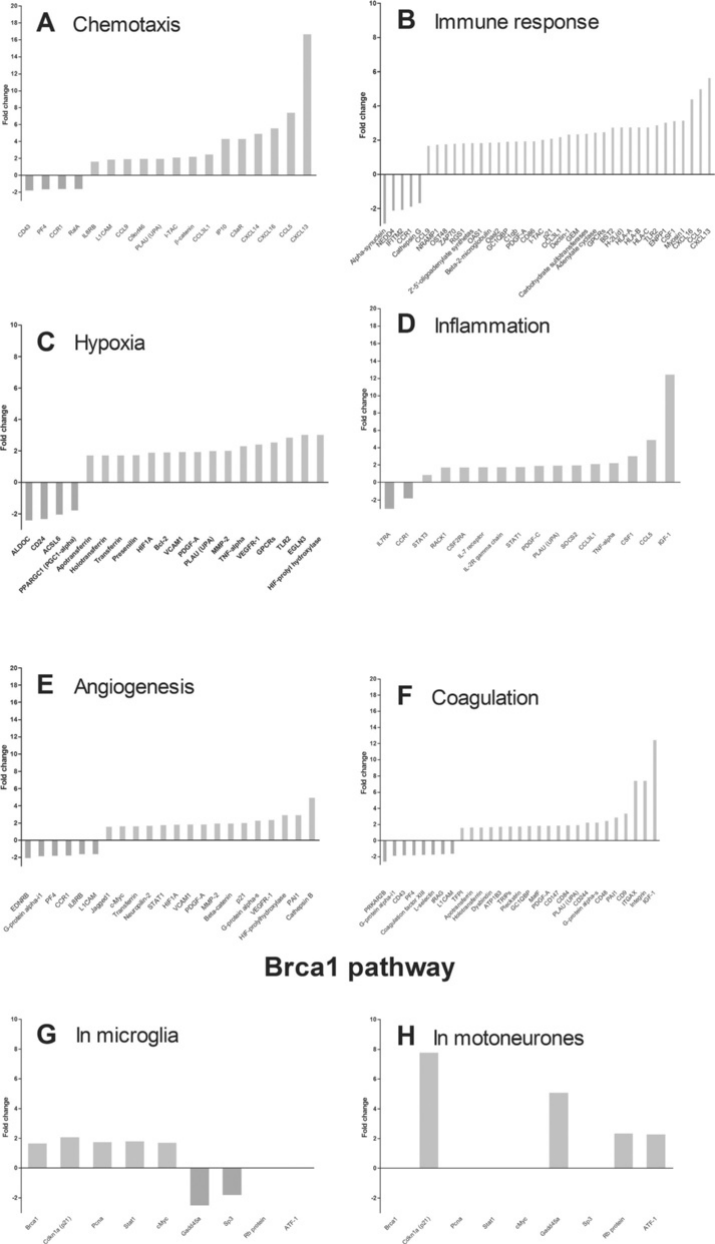
Enrichment and network analysis of dysregulated gene in pure hSOD1^G93A^ microglia at 90 days of age. Categories networks and gene ontology processes that are most significantly modified in hSOD1^G93A^ microglia at 90 days as compared to control microglia. In each category, the most dysregulated genes are presented

To identify processes and pathways modified in hSOD1^G93A^ microglia, we carried out gene ontology enrichment and network analysis (Additional file 5: Table S4A–C; Fig. 2). Process network analysis ranked as first chemotaxis (Additional file 5: Table S4A, Fig. 2a) with 23 dysregulated transcripts out of 137 annotated genes in this process (17 %, *p* = 2.1E-08) (Additional file 5: Table S4A). Out of the 18 most significantly dysregulated genes, 4 were down-regulated with a maximum of 2-fold whereas 14 were up-regulated (Fig. 2a). The gene coding for osteopontin (*SPP1*) presents a 16.8-fold increase (Additional file 1: Table S1). Regulation of angiogenesis (ranked 8th, Additional file 5: Table S4A, Fig. 2e) displayed 22 dysregulated genes (223 genes in this process, 9.8 %, *p* = 7.3E-04), with 16 being up-regulated. Inflammation network was also dysregulated in hSOD1^G93A^ microglia (ranked 9^th^, 9 % of the annotated genes in this process, *p* = 1.4E-04, Additional file 5: Table S4A, Fig. 2d) with 14 up-regulated genes (including a 12.6 fold increase for IGF-1, Fig. 2d). GO cellular processes analysis ranked immune response as first (61/1505 genes, 4 %, *p* = 1.3E-18, Additional file 5: Table S4B, Fig. 2b). Out of the 38 most significantly dysregulated genes 5 were down-regulated with a maximum of 2.97-fold decrease for the gene coding for alpha-synuclein, whereas 33 were up-regulated. Genes coding for CCL5 (5.1-fold change (FC)) and CXCL13 (5.7-FC) were the most up-regulated (Additional file 1: Table S1). Regulation of blood coagulation was ranked 3rd (50/665 genes, 7.5 %, *p* = 1.8E-09, Additional file 5: Table S4B, Fig. 2f). Amongst the 29 most significantly dysregulated genes 8 were down-regulated and 21 were up-regulated. Hypoxia was the 4th dysregulated cellular processes (31/416 genes, 7.45 %, *p* = 8.3E-09, Additional file 5: Table S4B). Out of the 20 most significantly dysregulated genes 4 were down-regulated and 16 were up-regulated (Fig. 2c).

### Breast cancer 1 (Brca1) pathway is dysregulated in hSOD1^G93A^ mice

**Fig. 3:**
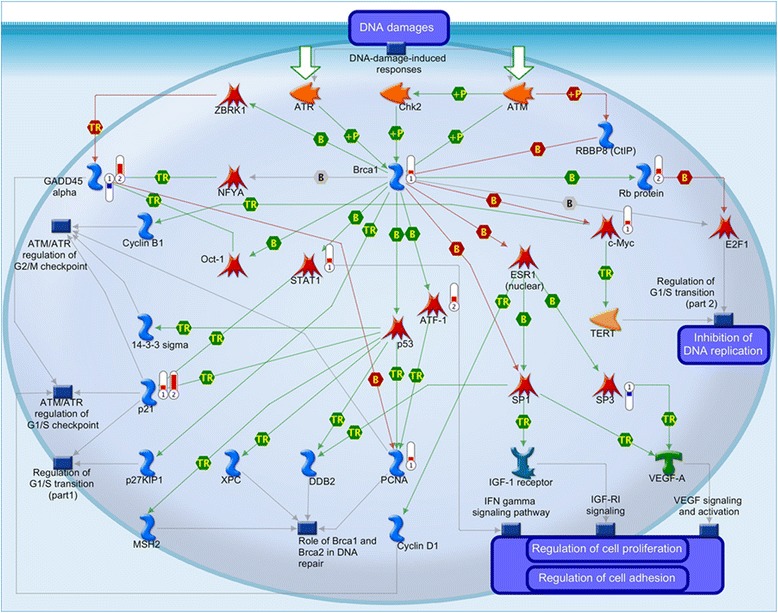
Brca1 pathway is involved in microglia from hSOD1^G93A^ mice. Gene ontology pathway map analyses of dysregulated genes either in hSOD1^G93A^ microglia at symptomatic age (P90) or in hSOD1^G93A^ motoneurones at the end stage of the disease (P120) demonstrate the involvement of Brca1 pathway. Thermometers indicate gene dysregulation (*red*: up-regulated; *blue*: down-regulated, thermometer levels correspond to the level of dysregulation). Thermometers with number *1* represent gene dysregulation in hSOD1^G93A^ microglia and number *2* in hSOD1^G93A^ motoneurones. Interactions between objects: *green* (positive or activation); *red* (negative or inhibition); *grey* (unspecified). *B* Binding (physical interaction between molecules), *TR* Transcription regulation (physical binding of a transcription factor to target gene’s promoter), +*p* Phosphorylation (protein activity is altered via addition of a phosphate group).  Binding protein  Transcription factor  Kinase  Generic enzyme

Pathways map analysis ranked as the third position DNA damage and specifically the involvement of Brca1 as a transcription regulator (Additional file 5: Table S4C, Figs. 2g and 3). Indeed, in hSOD1^G93A^ microglia (7/30 genes, 23 %; *p* = 1.6E-05, Additional file 5: Table S4C) were dysregulated in the canonical Brca1 pathway. *GADD45α* and *SP3* transcription factor were down-regulated with FC of 2.6 and 1.9, respectively. Genes coding for *p21* (2.18-FC), *PCNA* (1.85-FC), *STAT1* (1.9-FC), *c*-*Myc* (1.8-FC) and *Brca1* (1.76-FC) were up-regulated (Additional file 1: Table S1 and Fig. 2g). Concomitant dysregulation of these genes clearly pointed toward a potential involvement of *Brca1* as a transcription regulator (Fig. 2g and red and blue thermometers labelled in Fig. 3). Interestingly, even if *Brca1* transcript itself was not dysregulated in motoneurones, 4 genes that are involved in *Brca1* pathway were also up-regulated in hSOD1^G93A^ motoneurones namely *p21*: 7.88-FC; *GADD45α*: 5.19-FC; *Rb protein*: 2.44-FC and *ATF*-*1*: 2.38-FC, (Fig. 2h and red thermometers labelled 2 in Fig. 3). To confirm microarray findings, we carried out quantitative real-time polymerase chain reaction (qPCR) in pure populations of hSOD1^G93A^ and wild type microglia and assessed the expression profiles of all candidate genes involved in *Brca1* pathway (Additional file 6: Figure S2). In addition, we have also included microglial samples at 60 days of age to assess the potential involvement of microglial *Brca1* at the initial stages of the disease progression in hSOD1^G93A^ mice (Additional file 6: Figure S2A). Our qPCR results showed no significant dysregulation of the genes involved in *Brca1* pathway at 60 days of age (Additional file 6: Figure S2A). However, at 90 days of age, and similarly to our microarrays results, we found up-regulation of *Brca1*, *Cdkn1a*, *Myc*, *Pcna* and *Stat1* as well as down-regulation of *Gadd45a* and *Sp3* in hSOD1^G93A^ microglia (Additional file 6: Figure S2B). It is important to note that dysregulation in *Cdkn1a*, *Myc*, *Pcna*, *Stat1*, *Gadd45a* and *Sp3* transcripts may also be involved in other signalling pathways. These findings confirm *Brca1* involvement in hSOD1^G93A^ microglia is specifically triggered at 90 days of age when the pronounced microgliosis becomes evident.

### Brca1 protein is expressed in human microglia and is up-regulated in ALS patients

**Fig. 4:**
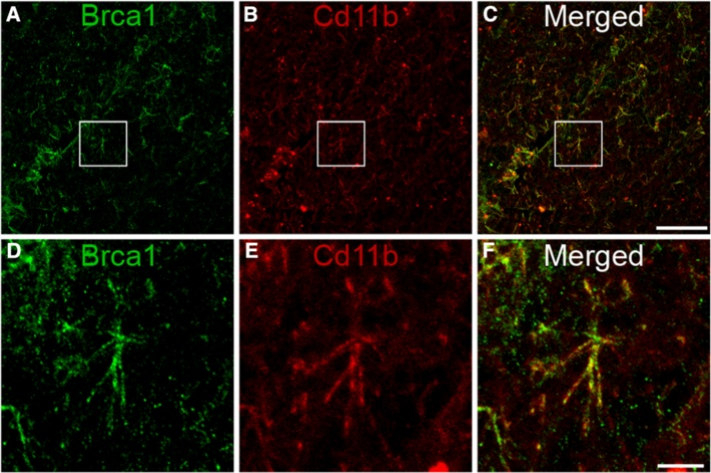
Microglial expression of Brca1 in human spinal cords. Confocal micrographs displaying Brca1 (**a**, **d**), CD11b (**b**, **e**) and dual Brca1/CD11b expression (**c**, **f**) expression by microglia within the human spinal cord. Brca1 labelled microglia displayed typical ramified morphology with small cell bodies and large and thin processes that completely colocalised with CD11b-positive microglia (**c & f**). *Scale bars* (**a**–**c**): 50 μm; (**d**–**f**): 10 μm

**Fig. 5:**
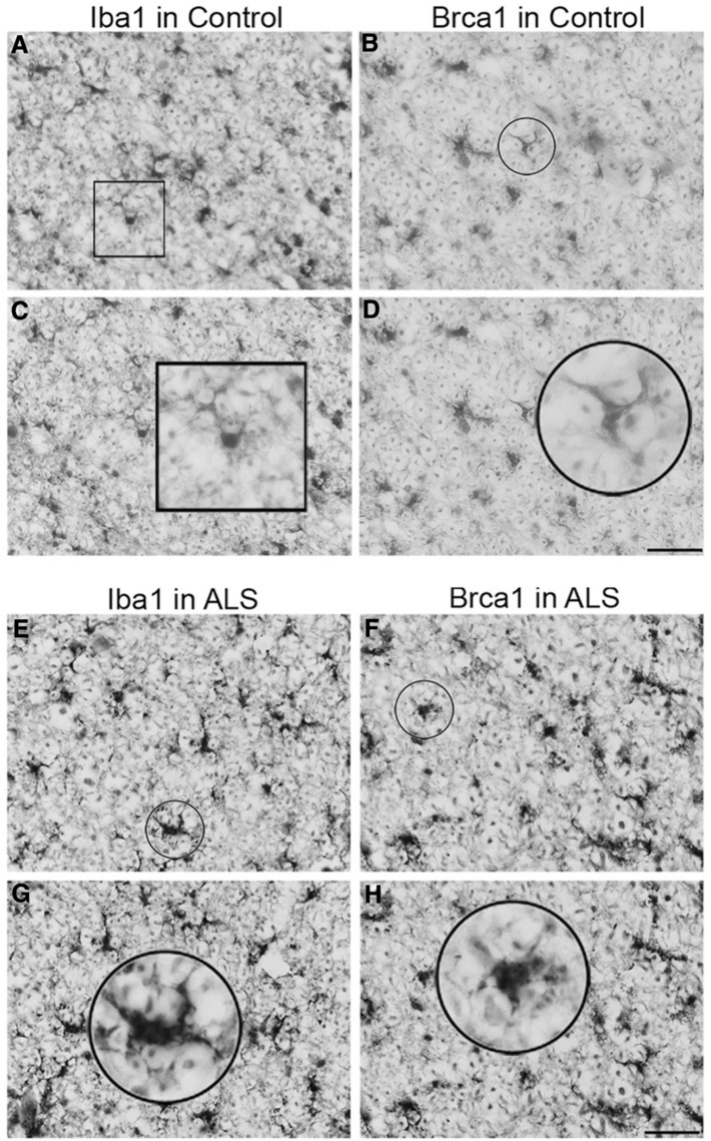
Microglial Iba1 and Brca1 expression within adjacent human control and ALS spinal cord. Brightfield micrographs indicating Iba1 and Brca1 expression within adjacent sections of control (**a**–**d**) and ALS (**e**–**h**) spinal cords. Both Ibal and Brca1-labelled microglia displayed typical ramified morphology in the control spinal cords with small cell bodies and large and thin processes (**c & d**). Microglial morphology displayed similar features following Iba1 (**a & c**) and Brca1 (**b & d**) immuno staining. In ALS spinal cords, Ibal and Brca1-labelled microglia displayed both ramified and activated microglia with enlarged cell bodies and short and thick processes (**g & h**). Microglial morphology were similar using Iba1 (**e & g**) or Brca1 (**f & h**) immuno staining. *Scale bars* (**a**–**h**): 50 μm

To investigate Brca1 protein expression in human microglia, we performed dual immunofluorescence labelling using Brca1 and CD11b antibodies (Fig. 4). Brca1 staining in human control samples revealed ramified microglial population throughout the spinal cord displaying small cell bodies with long and thin processes (Fig. 4a & d) that co-localised with CD11b-positive microglia (Fig. 4b & e, c & f). Similarly, single immunoperoxidase detection of Brca1 revealed microglial profile that were identical to Iba1 (the most commonly used microglia marker) using adjacent human spinal cord sections (Fig. 5). Ramified microglia were evident in control cases following both Brca1 and Iba1 immunoperoxidase labelling (Fig. 5a–d). On the other hand, Brca1-positive microglia displayed enlarged cell bodies with short/thick processes in ALS cases similar to Iba1 immunostaining (Fig. 5e–h).

**Fig. 6:**
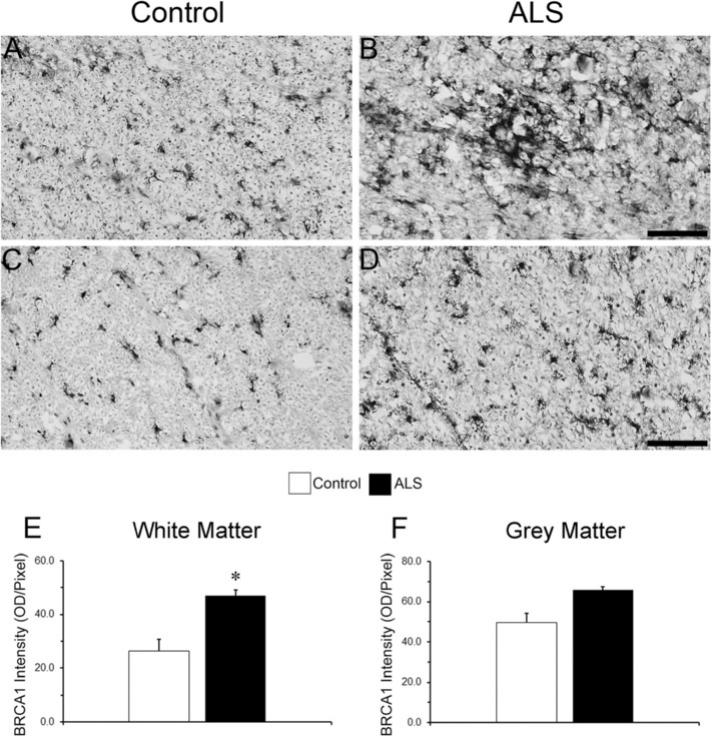
Up-regulation of microglial Brca1 expression in human ALS spinal cords. Brightfield micrographs displaying Brca1 expression within the spinal cords of control (**a & c**) and ALS cases (**b & d**). *Bar graphs* showing the increase in Brca1 intensity within the white (**e**) and grey (**f**) matters of the spinal cord in ALS compared to control cases. *Bars* represent mean ± SEM (*n* = 5 for ALS and 14 for controls). *p < 0.05. *Scale bar* (**a**–**d**): 100 μm

Finally, to determine Brca1 dysregulation in ALS, we quantified Brca1 immunoreactivity between the control and ALS spinal cords (Fig. 6). Brca1 expression was more evident in ALS compared to control cases (Fig. 6a & b, c & d). Quantitative analysis revealed a significant 78.2 % increase in Brca1 intensity within the white matter in ALS samples compared to controls (26.4 vs 47.1, *p* = 0.015; Fig. 6e). Within the grey matter, we observed a 32.8 % increase in Brca1 intensity in ALS samples compared to controls, however data variations in control samples kept them from attaining statistical significance (49.7 vs 66, *p* = 0.0545; Fig. 6f).

## Discussion

Non-cell autonomous toxicity plays a major role in ALS [20] but microglia participation is dual and complex. Microglia reactivity over the course of the disease may be characterised by a continuum of activation states from a M2 neuroprotective state to a deleterious M1 state. In culture, microglia have, at disease onset, a M2 phenotype whereas they are typified by a M1 phenotype at disease end-stage [21]. Comparison of our data to previous studies [21, 22] reveals an up-regulation of five M2 priming genes (*Clec7a*, *Igf1*, *Mmp12*, *Spp1* and *Lgals3*) and a down-regulation of *Retnla* and *F13a1*. Interestingly, four M1 priming genes are up-regulated *CD86*, *Tnfα*, *Bcl2a1*a and *Cxcl10*, whilst growth arrest and DNA-damage-inducible alpha gene (*Gadd45gip1*) is down-regulated. A previous study has reported the gene-expression profile of isolated microglia in hSOD1^G93A^ mice and shown that potentially neuroprotective and neurotoxic factors are induced concurrently during disease progression [23]. The authors have analysed microglia from the entire spinal cord whereas we have restricted our investigations to the lumbar segment where onset of degeneration occurs. We report that hSOD1^G93A^ microglia from the lumbar region of the spinal cord over-express *progranulin*, *Igf1* and *osteopontin*, all potential neurotrophic factors, and thus confirmed findings from a previous study [23]. Interestingly, we had previously identified in pure motoneurones of two mouse models of motoneurone disease (hSOD1^G93A^ and *pmn*) an increase in *IGFBP*. Also, an *IGFBP* that binds to *IGF*-*1* and *IGF*-*2* (nephroblastoma over-expressed gene) was up-regulated at all disease stages in hSOD1^G93A^ mice [8] and *IGFBP4* mRNA was induced at pre-symptomatic age in *pmn* mice [9]. We also confirmed the up-regulation of potential neurotoxic factors (including *Mmp12*, *tnf*-*α* and interferon-induced protein with tetratricopeptide repeats) [23]. However, we did not confirm the dysregulation of the genes coding for *IL*-*1β*, *IL*-*α*, *IL*-*10*, *Ifnar 1* and *Ifnar 2* as well as *Nox2* at P90. It had been shown that delayed forelimb motor impairment in ALS mice may be partially explained by augmented protective responses in the cervical spinal cords [24], thus gene expression profile of lumbar hSOD1^G93A^ microglia is potentially more homogenous and is more likely to reflect a pathological gene profile than microglia taken from the entire spinal cord. Together, these data confirm that microglia activation states are best characterised as a continuum of M2 and M1 states [21] with a M2 phenotype at early stage of the disease that evolves into a M1 phenotype at disease end-stage.

An unexpected finding was the up-regulation in hSOD1^G93A^ microglia of *Brca1* with a 1.76 fold. Using *in silico* comparison with data from Chiu et al. [23], we found that *Brca1* was also deregulated in their study and presented a steady increased with 2.78 and 3.08 fold changes at P100 and P130, respectively. In our study, *Brca1* involvement was substantiated by the concomitant dysregulation of a number of other genes. As previously stated, *Igf1* was robustly up-regulated in hSOD1^G93A^ microglia; a complex interplay between *Brca1* and *IGF* signalling pathways had been reported in familial cancer, in particular through the convergence of Brca1-mediated tumour protective pathways and IGF1 receptors-mediated cell survival [25, 26]. This simultaneous up-regulation may represent a potential neuroprotective phenotype of microglia in ALS at early stage. Converging elements toward the involvement of Brca1 was also pointed through the dysregulation of genes linked to *Brca1* and belonging to the DNA damage pathway (Fig. 3). Indeed, *GADD45* was down-regulated in hSOD1^G93A^ microglia (P90) and up-regulated in motoneurones (P120) and it had been demonstrated that *Brca1* can modulate *GADD45* that in turn mediates DNA repair mechanisms and regulates growth arrest [15]. Importantly, we found that the gene coding for cyclin-dependent kinase inhibitor 1A (*p21*) was up-regulated both in hSOD1^G93A^ microglia (P90) and motoneurones (P120). Indeed, *p21* is a downstream target of *p53* and regulates several processes such as DNA repair, cell cycle arrest, cell differentiation and apoptosis. Through its antioxidant effects, *p21* also protects cells from oxidative damage *in vitro* and* in vivo* [15]. Activation of microglial cells and acquisition of deleterious M1 state is associated with an increased generation of reactive oxygen species (ROS) [27] that is likely to participate in motoneurone demise. Polarisation of microglia/macrophages to pro- and anti-inflammatory states is driven by cytokines and other factors such as ROS within the tissue microenvironment [28]. While the functional role of deregulated *Brca1* pathway in microglia remains to be determined, one hypothesis is that it may represent an attempt to counteract the detrimental effects of ROS and reflect an antioxidative defence mechanism through modulation of microglia polarisation.

Brca1 is implicated in a broad spectrum of functions; it regulates transcription and cell cycle progression, it is also involved in function that preserve genomic stability such as DNA repair pathways [29] and protection against oxidative damage to DNA. Many of these functions have been associated with CNS development but also with neurodegenerative diseases and in particular with ALS. Brca1 is required for normal cerebral cortex size development [30] by preventing apoptosis [31]. Using a neural progenitor-specific driver to delete *Brca1*, Pao et al. demonstrated an important role of *Brca1* in apoptotic and centrosomal functions in neuronal progenitors that may underlie DNA damage and brain size during development [31]. *Brca1* is also associated with lack of spinal cord neural tube closure in* spina bifida* meningomyelocele [32, 33]. Moreover, *Brca1*-deficient embryos presented disorganised neuroepithelium associated with rapid proliferation and enhanced cell death [32].

De-regulation in *Brca1* expression had been reported in Alzheimer’s [34, 35] and Huntington’s diseases [36]. Even though motor neuron diseases are not typical paraneoplastic syndromes, association with breast cancer had been regularly reported [37–41]. Moreover, there are occasional reports on improvement of motor neuron syndrome after cancer treatment [42–44].

## Conclusion

Here we identify putative *Brca1* involvement in ALS via hSOD1^G93A^ microglia gene profiling and comparisons to our previous transcriptomic findings in hSOD1^G93A^ motoneurones. Nevertheless, mRNA up-regulation of *Brca1* in hSOD1^G93A^ microglia could be simply anecdotal if it were restricted to a mouse model of ALS. This is not the case since we demonstrated that Brca1 protein is specifically expressed by human microglia and is significantly up-regulated in ALS patients.

These results substantiate that microglia are key non-cell autonomous players in the disease. Thus, the identification of the putative Brca1 involvement in a mouse model and human ALS provides new insights into the pathogenesis of ALS and points towards novel therapeutic targets.

## Methods

### Animals

Transgenic mice carrying the G93A human SOD1 mutation, B6SJL-Tg (SOD1-G93A)1Gur/J (ALS mice, high copy number) were purchased from The Jackson Laboratory (Bar Harbor, ME, USA) and bred on a B6SJL background. Transgenic mice were housed in controlled conditions (hygrometry, temperature and 12 h light/dark cycle). Ninety days old (P90, symptomatic) males were used for transcriptomic analysis and immunohistochemistry. Litter-matching between groups were done. We carried out all animal experiments in accordance with the guidelines approved by the French Ministry of Agriculture and following the European Council directive (2010/63/UE). We minimised the number and suffering of animals.

### Flow cytometry sorting of spinal cord microglia from SOD^G93A^ and control littermate mice

Mice were deeply anesthetised with tribromoethanol (500 mg/kg) and intracardially perfused with cold RNAse-free 0.1 M phosphate base saline (PBS, Invitrogen, Carlsbad, USA); spinal cords were dissected. Only the lumbar (L1 - L5) segment was used and dissociated in 750 μl PBS, 100 μl trypsin 13 mg/ml, 100 μl hyaluronidase 7 mg/ml, 50 μl kinurenic acid 4 mg/ml (Sigma Aldrich, Saint Louis, USA) and 20 μl DNAseI 10 mg/ml (Roche, Rotkreuz, Switzerland) for 30 min at 37 °C. Finally, gentle mechanic dissociation was carried out by pipetting. Cell suspension was sieved on a 40 μm cell strainer (BD Biosciences, Franklin Lakes, USA). To eliminate myelin, cells were re-suspended in PBS-25 % sucrose and centrifuged for 20 min at 750 g. Cells were incubated for 20 min on ice in the primary antibody CD11b-APC 1/100 in PBS (BD biosciences, Franklin Lakes, USA) that specifically labels microglia. Cells were washed with cold PBS and re-suspended in PBS 7-AAD 2 μg/ml (Sigma Aldrich). Cells were sorted with a FACS ARIA (BD Biosciences, Franklin Lakes, USA), equipped with a 488 nm Laser Sapphire 488–20. Size threshold, morphology and 7-AAD were used to eliminate cellular debris and dead cells.

### Microarray analysis of gene transcripts

Our data comply with the “Minimal Information About Microarray Experiment (MIAME)” guidelines. Total RNA was isolated using RNeasy Mini Kit, (Qiagen, Maryland, USA) including DNAse treatment to remove potential genomic DNA contamination. We tested the quality of the starting RNA and of the amplified cRNA (Agilent 2100 bioanalyzer, RNA 6000 Pico LabChip, Palo Alto, USA) and proceeded only if the RNA quality was satisfactory. A criterion was a cut point for RNA integrity number (RIN) at 7 [45]. Fifty nanograms of RNA per chip were hybridized (three chips per condition) following a T7-based double amplification procedure.

Hybridization targets were obtained following a double amplification procedure according to the protocol developed by Affymetrix (GeneChip® Eukaryotic Small Sample Target Labeling Assay Version II, Affymetrix, Santa Clara, USA) and previously used [8, 9]. A hybridization mixture containing 5.5 μg of biotinylated cRNA was generated. The biotinylated cRNA was hybridized to Affymetrix GeneChip® MOE 430 2.0. Three chips per group (wild type and hSOD1^G93A^) were hybridized, each corresponding to microglia from at least six pooled mice. Chips were visualised on a 3000 gene scanner (Affymetrix, Santa Clara, USA). We selected the differentially expressed transcripts using the Affymetrix software MAS 5.0 and carried out pair-wise comparison analyses where each of the mutant samples was compared to each of their respective control samples. This analysis is based on the Mann–Whitney pair-wise comparison test and allows the ranking of the results by concordance as well as the calculation of significance (*p* value) of each identified gene expression [46, 47]. A gene must exhibit 50 % or more of the “present” calls in all samples to be considered “expressed” and has two or more “present” calls among the three sets of samples. Fold differences were calculated as the ratio between the average values within each condition. Signal values and detection calls (present or absent) for all samples were determined using Affymetrix MAS5.0. Based on power analysis, we had selected a cut off threshold of 1.75 (p (α) 0.05, β 0.80) to identify transcripts that are differentially expressed between the controls and hSOD1^G93A^ mutant mice. Statistics: t-test with un-equal variance. Pathway analysis was done with MetaCore (Thomson Reuters).

### Quantitative real-time polymerase chain reaction

Candidate genes involved in Brca1 pathway were validated using qPCR. Similar to microarray, total RNA was extracted as described above from CD11-positive microglia isolated using FACS and used as a template in real time PCR. At least two animals were used for each analysis. To assess the involvement of microglia *Brca1* at initial stages of the disease progression, we carried out qPCR at 60 and 90 days of age in hSOD1^G93A^ and wild type mice. One round of amplification was done following the first cycle (first cDNA and cRNA synthesis) of the Affymetrix double amplification procedure before undertaking reverse transcription with random hexamers (Superscript II, Invitrogen, Carlsbad, CA). Real time PCR using Syber Green PCR Master Mix and Abi Prism SDS 7900 HT (Applied Biosystems, Foster City, CA) was done according to the manufacturer’s protocol. All amplicons were designed within the 3′ end of the cDNA using Primer Express Software 2.0 (Applied Biosystems, Foster City, CA) and when possible, overlapped exon-exon junctions. For the sequences of the primers, see Additional file 7: Table S5. All samples were analysed in triplicate and the values were normalised to four reference genes mitochondrial ribosomal protein S9 (*RPS9*), TATA box binding protein (*TBP*), actin β and eukaryotic translation elongation factor 1 (*EEF1*).

### Human spinal cord samples

Human low thoracic and lumbar (T11-L5) spinal cords were obtained from 14 controls (males and females; 23 to 74 years of age; mean age: 52.4 years) and five ALS patients (males and females; 66 to 79 years of age; mean age: 71 years) from the Kantonsspital St. Gallen Fachbereichsleiter Muskelzentrum/ALS clinic under the approval of the Swiss legislation and from the New York Brain Bank–Taub Institute, Columbia University (NYBB), New York, USA. All donors had given their written consent for the autopsy and we followed the Declaration of Helsinki.

### Immunohistochemistry

Mice were anesthetised with tribromoethanol (500 mg/kg) and perfused intracardially with cold PBS followed by cold 4 % paraformaldehyde (PFA, Sigma Aldrich). Spinal cords were removed and post fixed for 2 h in 4 % PFA. Samples were cryoprotected in sucrose 30 %, included in Tissue Teck (Sakura, Alphen aan den Rijn, The Netherlands), frozen and kept at −80 °C until processing.

For mice, free floating spinal cord transverse sections (20 μm) were washed twice in PBS (5 min), treated for 30 min in PBS containing lysine (20 mM, pH 7.2) and for 15 min in 1 % H_2_O_2_. Sections were blocked for 1 h with PBS containing bovine serum albumin (BSA, 1 %, Sigma Aldrich) and Triton X-100 (0.1 %, Fisher Scientific, Illkirch, France) and then incubated 48 h at 4 °C with CD11b (1/200, Developmental Studies Hybridoma Bank, Iowa, USA) primary antibody. Alexa-conjugated 594 secondary antibody was used (1/1000; Molecular Probes, Eugene, OR, USA).

For human spinal cord 22 μm-thick cryosections of lumbar and lower thoracic segments were collected on super frost plus slides and were processed as described above. For dual fluorescence labelling, sections were placed in a cocktail of rat anti CD11b (1/100, Hybridoma Bank, University of Iowa, USA) and rabbit anti-Brca1 (1/100, Santa Cruz Biotechnology, Dallas, USA) primary antibodies for 48 h at 4 °C. Sections were washed in 0.1 M PBS followed by incubation in corresponding secondary antibodies conjugated to Alexa 488 and 594 (1/1000; Molecular Probes, Eugene, OR, USA). For peroxidase labelling, sections were placed for 48 h at 4 °C in either rabbit anti Iba1 (macrophage/microglia-specific calcium-binding protein) (1/1000, Wako Pure Chemical Industries, Osaka, Japan) or rabbit anti-Brca1 (1/100, Santa Cruz Biotechnology, Dallas, USA) primary antibodies. Spinal cord sections were then incubated in donkey anti-rabbit (1/500, Jackson Immunoresearch, Carlsbad, USA) antibody for 2 h at 4 °C. Sections were then washed in TRIS buffer and enzymatic revelation was done with nickel enhanced DAB and H_2_O_2_ 0.1 % as a substrate. Sections were then dehydrated in ascending concentration of ethanol and finally xylene. Coverslips were applied using Entellan (Merck KGaA, Darmstadt, Germany).

Morphometric bright field photographs had been obtained and analysed using NanoZoomer RS slide scanner (NanoZoomer Digital Pathology System and NDP view software, Hamamatsu, Japan). For immunofluorescence images, we used laser scanning inverted confocal microscopy (Leica SP5, Mannheim, Germany). Laser intensity and detector sensitivity settings were kept constant for all image acquisitions within a given experiment. Brca1 staining intensity measurement was done by measuring their optical density (OD) using ImageJ (National Institutes of Health, USA), as described previously [48]. For each given sample we analysed at least three 22-μm-thick section with 330 μm distance from each other. Statistics: un-paired t-test done with GraphPad Prism version 5.03 (GraphPad software, CA, USA). Significance was accepted at *p* ≤ 0.05. Results are expressed as mean ± S.E.M.

## Additional files

Additional file 1: Table S1. Database of differential expression comparison of hSOD1^G93A^ microglia microarray data relative to control microglia at 90 days of age. We list information for each dysregulated genes in hSOD1^G93A^ microglia as compared to control microglia. With both the *p*-value and the step-up *p*-value that is the false discovery rate (FDR) analogue of the *p*-value. Three chips were used per condition (wild type and SOD1^G93A^) with microglia from lumbar spinal cord of at least six pooled mice. Additional file 2: Figure S1. Comparison of gene dysregulation in microglia and motoneurones. (A) Venn diagrams showing that 19 genes are commonly dysregulated in microglia and motoneurones at symptomatic age (P90). (B) Comparison of dysregulated genes in microglia at P90 and motoneurones at disease end stage shows that 65 genes are commonly dysregulated, 603 genes are uniquely dysregulated in hSOD1^G93A^ microglia and 320 uniquely in hSOD1^G93A^ motoneurones. Three chips were used per condition (wild type and SOD1^G93A^) with microglia from lumbar spinal cord of at least six pooled mice. Additional file 3: Table S2. Comparison of gene dysregulation in both microglia and motoneurones at symptomatic age (P90) and in motoneurone only (P90) using gene ontology enrichment and network analysis. In all tables the top scored categories have the lowest *p*-value. Table S2A: Process networks ranking. Table S2B: Gene ontology processes ranking and Table S2C: Pathway maps ranking. Additional file 4: Table S3. Comparison of gene dysregulation in microglia at symptomatic age (P90) and in motoneurone at the end stage of the disease (P120) using gene ontology enrichment and network analysis. In all tables the top scored categories have the lowest *p*-value. Table S3A: Process networks ranking. Table S3B: Gene ontology processes ranking and Table S3C: Pathway maps ranking. Additional file 5: Table S4. Gene ontology enrichment and network analysis of gene dysregulation in microglia at symptomatic age (P90). In all tables the top scored categories have the lowest *p*-value. Table S4A: Process networks. Table S4B: Gene ontology processes and Table S4C: Pathway maps. Percentage of dysregulated genes corresponds to the ratio of dysregulated genes in our data out of annotated genes in the given category (Gene Ontology). Additional file 6: Figure S2. Quantitative real-time polymerase chain reaction (qPCR) validation of microarray findings related to candidate genes involved in *Brca1* pathway. To confirm the microarray results, the seven identified genes involved in *Brca1* pathway were analysed by real time qPCR. Bar graphs showing up-regulation of *Brca1*, *Cdkn1a*, *Myc*, *Pcna* and *Stat1* as well as down-regulation of *Gadd45a* and *Sp3* in hSOD1^G93A^ microglia at 90 (B) but not 60 days (A) as compared to control microglia. For each sample, real time PCR was done in triplicate. Additional file 7: Table S5. Candidate genes involved in Brca1 pathway selected for qPCR to validate the microarray data.

## Abbreviations

ALS: Amyotrophic lateral sclerosis
CNS: Central nervous system
DAB: Diaminobenzidine
ER: Endoplasmic reticulum
FACS: Fluorescence-activated cell sorting
FALS: Familial amyotrophic lateral sclerosis
FC: Fold change
GO: Gene ontology
OD: Optical density
PBS: Phosphate base saline
PFA: Paraformaldehyde
pmn: Progressive motor neuronopathy
RIN: RNA integrity number
ROS: Reactive oxygen species
SOD: Super oxide dismutase
