# Supplementary figures and images for: Brca1 is expressed in human microglia and is dysregulated in human and animal model of ALS
Source: Mol Neurodegener. 2015 Aug 1;10:34. doi: 10.1186/s13024-015-0023-x (PMC4521418; doi:10.1186/s13024-015-0023-x)

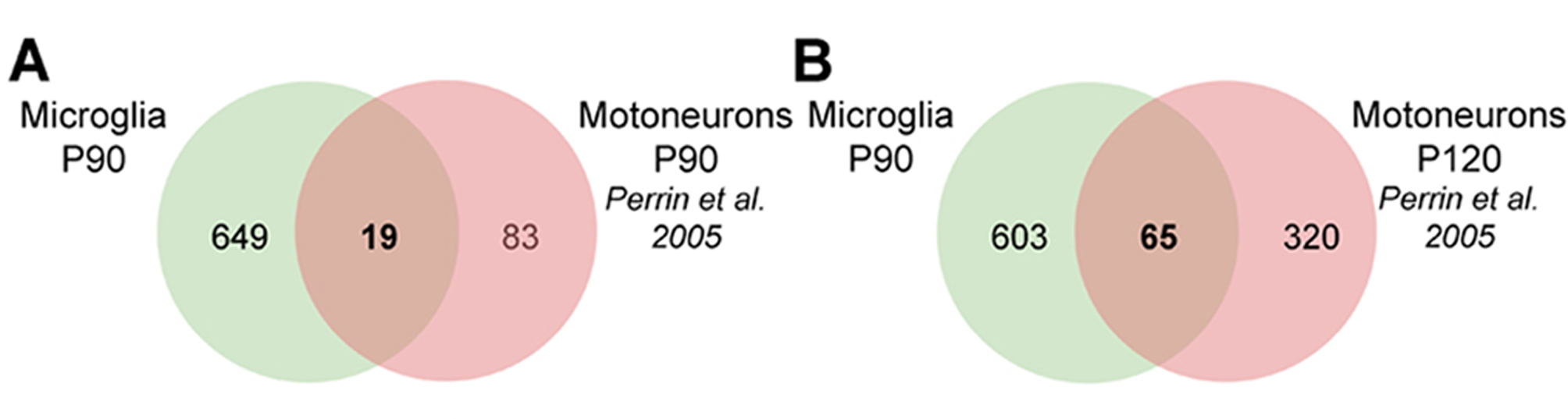

Supplement: Additional file 1: Table S1. — Database of differential expression comparison of hSOD1G93A microglia microarray data relative to control microglia at 90 days of age. We list information for each dysregulated genes in hSOD1G93A microglia as compared to control microglia. With both the p-value and the step-up p-value that is the false discovery rate (FDR) analogue of the p-value. Three chips were used per condition (wild type and SOD1G93A) with microglia from lumbar spinal cord of at least six pooled mice. [file 13024_2015_23_MOESM1_ESM.tiff]

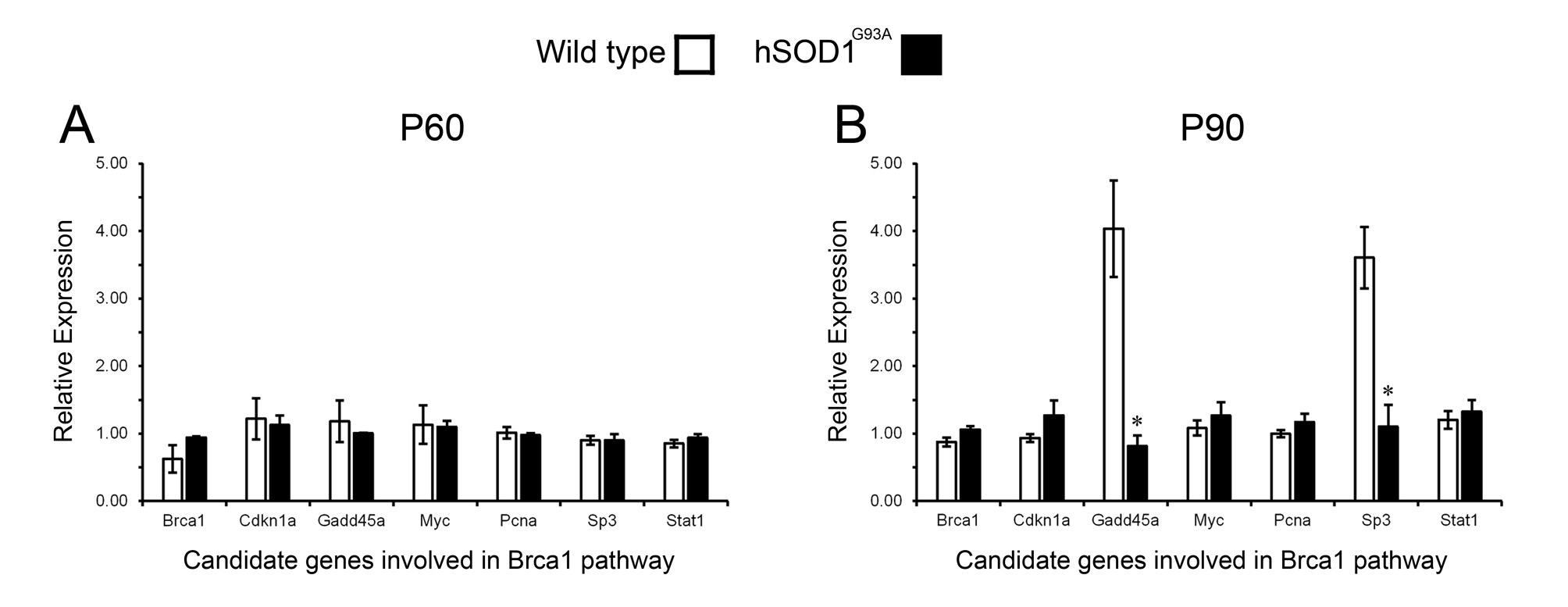

Supplement: Additional file 6: Figure S2. — Quantitative real-time polymerase chain reaction (qPCR) validation of microarray findings related to candidate genes involved in Brca1 pathway. To confirm the microarray results, the seven identified genes involved in Brca1 pathway were analysed by real time qPCR. Bar graphs showing up-regulation of Brca1, Cdkn1a, Myc, Pcna and Stat1 as well as down-regulation of Gadd45a and Sp3 in hSOD1G93A microglia at 90 (B) but not 60 days (A) as compared to control microglia. For each sample, real time PCR was done in triplicate. [file 13024_2015_23_MOESM6_ESM.tiff]
