# Supplementary material for: Brca1 is expressed in human microglia and is dysregulated in human and animal model of ALS
Source: Mol Neurodegener. 2015 Aug 1;10:34. doi: 10.1186/s13024-015-0023-x (PMC4521418; doi:10.1186/s13024-015-0023-x)
Supplement: Additional file 3: Table S2. — Comparison of gene dysregulation in both microglia and motoneurones at symptomatic age (P90) and in motoneurone only (P90) using gene ontology enrichment and network analysis. In all tables the top scored categories have the lowest p-value. Table S2A: Process networks ranking. Table S2B: Gene ontology processes ranking and Table S2C: Pathway maps ranking. [file 13024_2015_23_MOESM3_ESM.docx]

**Supplementary Table 2A : Process networks ranking**

Cellular and molecular processes rank pre-set common network of protein interactions that are significantly deregulated in both hSOD1^G93A^ motoneurone and microglia as compared to their respective controls and significantly deregulated only in hSOD1^G93A^ motoneurone. For both cell populations transcriptomic was done at symptomatic age (P90). The top scored process has the lowest p-value.

| **Rank** | **Process networks analysis in both motoneurones and microglia** | **P value** | **Process networks analysis motoneurones only** | **P value** |
| --- | --- | --- | --- | --- |
| **1** | [Immune response_Antigen presentation](http://portal.genego.com/cgi/network/net_net.cgi?term=10&id=145147) | 2.146E-03 | [Cytoskeleton_Cytoplasmic microtubules](http://portal.genego.com/cgi/network/net_net.cgi?term=10&id=145085) | 4.116E-04 |
| **2** | [Immune response_Phagosome in antigen presentation](http://portal.genego.com/cgi/network/net_net.cgi?term=10&id=145109) | 3.903E-03 | [Cytoskeleton_Intermediate filaments](http://portal.genego.com/cgi/network/net_net.cgi?term=10&id=145146) | 5.981E-04 |
| **3** | [Reproduction_Progesterone signaling](http://portal.genego.com/cgi/network/net_net.cgi?term=10&id=145098) | 3.429E-02 | [Cell adhesion_Cell-matrix interactions](http://portal.genego.com/cgi/network/net_net.cgi?term=10&id=145125) | 1.996E-03 |
| **4** | [Cytoskeleton_Intermediate filaments](http://portal.genego.com/cgi/network/net_net.cgi?term=10&id=145146) | 1.078E-01 | [Signal transduction_Leptin signaling](http://portal.genego.com/cgi/network/net_net.cgi?term=10&id=145187) | 2.095E-03 |
| **5** | [Cell cycle_Meiosis](http://portal.genego.com/cgi/network/net_net.cgi?term=10&id=145212) | 1.389E-01 | [Cell adhesion_Integrin-mediated cell-matrix adhesion](http://portal.genego.com/cgi/network/net_net.cgi?term=10&id=145130) | 2.163E-03 |
| **6** | [Cytoskeleton_Spindle microtubules](http://portal.genego.com/cgi/network/net_net.cgi?term=10&id=145164) | 1.426E-01 | [Cytoskeleton_Regulation of cytoskeleton rearrangement](http://portal.genego.com/cgi/network/net_net.cgi?term=10&id=145201) | 4.487E-03 |
| **7** | [Cytoskeleton_Cytoplasmic microtubules](http://portal.genego.com/cgi/network/net_net.cgi?term=10&id=145085) | 1.498E-01 | [Reproduction_Progesterone signaling](http://portal.genego.com/cgi/network/net_net.cgi?term=10&id=145098) | 9.473E-03 |
| **8** | [Proteolysis_Connective tissue degradation](http://portal.genego.com/cgi/network/net_net.cgi?term=10&id=145203) | 1.547E-01 | [Signal transduction_Neuropeptide signaling pathways](http://portal.genego.com/cgi/network/net_net.cgi?term=10&id=145097) | 1.009E-02 |
| **9** | [Proteolysis_Proteolysis in cell cycle and apoptosis](http://portal.genego.com/cgi/network/net_net.cgi?term=10&id=145701) | 1.619E-01 | [Cytoskeleton_Spindle microtubules](http://portal.genego.com/cgi/network/net_net.cgi?term=10&id=145164) | 1.373E-02 |
| **10** | [Cell adhesion_Cell junctions](http://portal.genego.com/cgi/network/net_net.cgi?term=10&id=145075) | 2.050E-01 | [Reproduction_Gonadotropin regulation](http://portal.genego.com/cgi/network/net_net.cgi?term=10&id=145171) | 2.689E-02 |

**Supplementary Table 2B : Gene ontology processes ranking**

Gene ontology (GO) processes rank cellular processes that are significantly deregulated in both hSOD1^G93A^ motoneurone and microglia as compared to their respective controls and significantly deregulated only in hSOD1^G93A^ motoneurone. For both cell populations transcriptomic was done at symptomatic age (P90). The top scored process has the lowest p-value.

| **Rank** | **GO processes analysis in both motoneurones and microglia** | **P value** | **GO processes analysis in motoneurones only** | **P value** |
| --- | --- | --- | --- | --- |
| **1** | [Antigen processing and presentation of endogenous peptide antigen via MHC class I via ER pathway, TAP-dependent](http://portal.genego.com/cgi/process.cgi?id=-849382407) | 1.224E-09 | [Response to stress](http://portal.genego.com/cgi/process.cgi?id=-1767027701) | 4.834E-12 |
| **2** | [Antigen processing and presentation of endogenous peptide antigen via MHC class I via ER pathway](http://portal.genego.com/cgi/process.cgi?id=-736090939) | 1.224E-09 | [System development](http://portal.genego.com/cgi/process.cgi?id=-2069873154) | 4.253E-10 |
| **3** | [Antigen processing and presentation of endogenous peptide antigen via MHC class I](http://portal.genego.com/cgi/process.cgi?id=-1868458523) | 3.070E-09 | [Positive regulation of immune response](http://portal.genego.com/cgi/process.cgi?id=-1502902269) | 6.370E-10 |
| **4** | [Antigen processing and presentation of endogenous peptide antigen](http://portal.genego.com/cgi/process.cgi?id=-866157694) | 5.441E-09 | [Response to wounding](http://portal.genego.com/cgi/process.cgi?id=-456070347) | 2.284E-09 |
| **5** | [Antigen processing and presentation of endogenous antigen](http://portal.genego.com/cgi/process.cgi?id=-269943210) | 7.646E-09 | [Defense response](http://portal.genego.com/cgi/process.cgi?id=-1137554385) | 2.349E-09 |
| **6** | [Positive regulation of T cell mediated cytotoxicity](http://portal.genego.com/cgi/process.cgi?id=-1939928572) | 2.445E-07 | [Innate immune response](http://portal.genego.com/cgi/process.cgi?id=-866848204) | 2.464E-09 |
| **7** | [Regulation of T cell mediated cytotoxicity](http://portal.genego.com/cgi/process.cgi?id=-2054036869) | 4.570E-07 | [Multicellular organismal process](http://portal.genego.com/cgi/process.cgi?id=-404497830) | 7.017E-09 |
| **8** | [Positive regulation of T cell mediated immunity](http://portal.genego.com/cgi/process.cgi?id=-1718919485) | 6.718E-07 | [Immune response](http://portal.genego.com/cgi/process.cgi?id=-195107408) | 8.370E-09 |
| **9** | [Positive regulation of leukocyte mediated cytotoxicity](http://portal.genego.com/cgi/process.cgi?id=-1549678166) | 8.240E-07 | [Single-multicellular organism process](http://portal.genego.com/cgi/process.cgi?id=-1584729523) | 1.537E-08 |
| **10** | [Positive regulation of cell killing](http://portal.genego.com/cgi/process.cgi?id=-985970372) | 1.099E-06 | [Multicellular organismal development](http://portal.genego.com/cgi/process.cgi?id=-528578425) | 2.171E-08 |

**Supplementary Table 2C : Pathway maps ranking**

Canonical pathway maps rank signaling and metabolic maps that are significantly in both hSOD1^G93A^ motoneurone and microglia as compared to their respective controls and significantly deregulated only in hSOD1^G93A^ motoneurone. For both cell populations transcriptomic was done at symptomatic age (P90). The top scored process has the lowest p-value.

| **Rank** | **Pathway maps analysis in both motoneurones and microglia** | **P value** | **Pathway maps analysis in motoneurones only** | **P value** |
| --- | --- | --- | --- | --- |
| **1** | [Immune response_Antiviral actions of Interferons](http://portal.genego.com/cgi/imagemap.cgi?id=2225) | 1.117E-03 | Cytoskeleton remodeling_Neurofilaments | 4.762E-08 |
| **2** | [Glycine, serine, cysteine and threonine metabolism](http://portal.genego.com/cgi/imagemap.cgi?id=800) | 5.905E-03 | Cytoskeleton remodeling_Keratin filaments | 1.368E-05 |
| **3** | [Glycine, serine, cysteine and threonine metabolism/ Rodent version](http://portal.genego.com/cgi/imagemap.cgi?id=2311) | 6.096E-03 | Transcription_Transcription regulation of aminoacid metabolism | 1.438E-04 |
| **4** | [Schema: Initiation of T cell recruitment in allergic contact dermatitis](http://portal.genego.com/cgi/imagemap.cgi?id=5078) | 1.762E-02 | Reproduction_GnRH signaling | 2.146E-04 |
| **5** | [Proteolysis_Role of Parkin in the Ubiquitin-Proteasomal Pathway](http://portal.genego.com/cgi/imagemap.cgi?id=662) | 2.343E-02 | Cell adhesion_Gap junctions | 2.502E-04 |
| **6** | [Cytoskeleton remodeling_Neurofilaments](http://portal.genego.com/cgi/imagemap.cgi?id=1491) | 2.439E-02 | Cell cycle_Role of Nek in cell cycle regulation | 3.039E-04 |
| **7** | [Development_Role of CNTF and LIF in regulation of oligodendrocyte development](http://portal.genego.com/cgi/imagemap.cgi?id=4787) | 2.729E-02 | Immune response_Oncostatin M signaling via MAPK in mouse cells | 3.975E-04 |
| **8** | [Immune response_Antigen presentation by MHC class I](http://portal.genego.com/cgi/imagemap.cgi?id=2100) | 2.729E-02 | Immune response_Oncostatin M signaling via MAPK in human cells | 4.692E-04 |
| **9** | [Cell adhesion_Gap junctions](http://portal.genego.com/cgi/imagemap.cgi?id=1485) | 2.921E-02 | Development_Beta-adrenergic receptors transactivation of EGFR | 4.692E-04 |
| **10** | [Cell cycle_Role of Nek in cell cycle regulation](http://portal.genego.com/cgi/imagemap.cgi?id=731) | 3.113E-02 | Development_Astrocyte differentiation from adult stem cells | 5.916E-04 |
