# Supplementary material for: Brca1 is expressed in human microglia and is dysregulated in human and animal model of ALS
Source: Mol Neurodegener. 2015 Aug 1;10:34. doi: 10.1186/s13024-015-0023-x (PMC4521418; doi:10.1186/s13024-015-0023-x)
Supplement: Additional file 4: Table S3. — Comparison of gene dysregulation in microglia at symptomatic age (P90) and in motoneurone at the end stage of the disease (P120) using gene ontology enrichment and network analysis. In all tables the top scored categories have the lowest p-value. Table S3A: Process networks ranking. Table S3B: Gene ontology processes ranking and Table S3C: Pathway maps ranking. [file 13024_2015_23_MOESM4_ESM.docx]

**Supplementary Table 3A : Process networks ranking**

Cellular and molecular processes rank pre-set common network of protein interactions that are significantly deregulated in both hSOD1^G93A^ motoneurone and microglia as compared to their respective controls and significantly deregulated only in hSOD1^G93A^ motoneurone. Transcriptomic was done at symptomatic age (P90) in microglia and at the end stage of the disease (P120) for motoneurones. The top scored process has the lowest p-value.

| **Rank** | **Process Networks**  **(both motoneurones and microglia)** | **P value** | **Process Networks**  **(motoneurones only)** | **P value** |
| --- | --- | --- | --- | --- |
| **1** | [Transport_Iron transport](http://portal.genego.com/cgi/network/net_net.cgi?term=10&id=145172) | 2.220E-03 | [Inflammation_Complement system](http://portal.genego.com/cgi/network/net_net.cgi?term=10&id=145133) | 8.892E-07 |
| **2** | [Immune response_Antigen presentation](http://portal.genego.com/cgi/network/net_net.cgi?term=10&id=145147) | 3.219E-03 | [Immune response_Phagocytosis](http://portal.genego.com/cgi/network/net_net.cgi?term=10&id=145169) | 4.970E-06 |
| **3** | [Cell adhesion_Leucocyte chemotaxis](http://portal.genego.com/cgi/network/net_net.cgi?term=10&id=145115) | 3.819E-03 | [Reproduction_FSH-beta signaling pathway](http://portal.genego.com/cgi/network/net_net.cgi?term=10&id=145088) | 5.018E-05 |
| **4** | [Immune response_Phagosome in antigen presentation](http://portal.genego.com/cgi/network/net_net.cgi?term=10&id=145109) | 7.814E-03 | [Cell adhesion_Integrin-mediated cell-matrix adhesion](http://portal.genego.com/cgi/network/net_net.cgi?term=10&id=145130) | 1.288E-04 |
| **5** | [Reproduction_Feeding and Neurohormone signaling](http://portal.genego.com/cgi/network/net_net.cgi?term=10&id=145150) | 2.278E-02 | [Cytoskeleton_Regulation of cytoskeleton rearrangement](http://portal.genego.com/cgi/network/net_net.cgi?term=10&id=145201) | 2.295E-04 |
| **6** | [Cell adhesion_Cell-matrix interactions](http://portal.genego.com/cgi/network/net_net.cgi?term=10&id=145125) | 2.278E-02 | [Cytoskeleton_Actin filaments](http://portal.genego.com/cgi/network/net_net.cgi?term=10&id=145194) | 5.040E-04 |
| **7** | [Reproduction_Progesterone signaling](http://portal.genego.com/cgi/network/net_net.cgi?term=10&id=145098) | 2.385E-02 | [Cell adhesion_Cell-matrix interactions](http://portal.genego.com/cgi/network/net_net.cgi?term=10&id=145125) | 1.037E-03 |
| **8** | [Inflammation_Neutrophil activation](http://portal.genego.com/cgi/network/net_net.cgi?term=10&id=145341) | 2.421E-02 | [Inflammation_Amphoterin signaling](http://portal.genego.com/cgi/network/net_net.cgi?term=10&id=145138) | 1.992E-03 |
| **9** | [Development_Blood vessel morphogenesis](http://portal.genego.com/cgi/network/net_net.cgi?term=10&id=145160) | 2.925E-02 | [Inflammation_IL-6 signaling](http://portal.genego.com/cgi/network/net_net.cgi?term=10&id=145174) | 2.122E-03 |
| **10** | [Inflammation_MIF signaling](http://portal.genego.com/cgi/network/net_net.cgi?term=10&id=145197) | 3.521E-02 | [Cytoskeleton_Intermediate filaments](http://portal.genego.com/cgi/network/net_net.cgi?term=10&id=145146) | 2.136E-03 |

**Supplementary Table 3B : Gene ontology processes ranking**

Gene ontology (GO) processes rank cellular processes that are significantly deregulated in both hSOD1^G93A^ motoneurone and microglia as compared to their respective controls and significantly deregulated only in hSOD1^G93A^ motoneurone. Transcriptomic was done at symptomatic age (P90) in microglia and at the end stage of the disease (P120) for motoneurones. The top scored process has the lowest p-value.

| **Rank** | **GO Processes**  **(both motoneurones and microglia)** | **P value** | **GO Processes**  **(motoneurones only)** | **P value** |
| --- | --- | --- | --- | --- |
| **1** | [Antigen processing and presentation of endogenous peptide antigen via MHC class I via ER pathway, TAP-dependent](http://portal.genego.com/cgi/process.cgi?id=-849382407) | 3.382E-14 | [Response to stress](http://portal.genego.com/cgi/process.cgi?id=-1767027701) | 2.174E-26 |
| **2** | [Antigen processing and presentation of endogenous peptide antigen via MHC class I via ER pathway](http://portal.genego.com/cgi/process.cgi?id=-736090939) | 3.382E-14 | [Positive regulation of biological process](http://portal.genego.com/cgi/process.cgi?id=-135855955) | 8.428E-22 |
| **3** | [Antigen processing and presentation of endogenous peptide antigen via MHC class I](http://portal.genego.com/cgi/process.cgi?id=-1868458523) | 2.003E-13 | [Response to wounding](http://portal.genego.com/cgi/process.cgi?id=-456070347) | 9.908E-20 |
| **4** | [Antigen processing and presentation of endogenous peptide antigen](http://portal.genego.com/cgi/process.cgi?id=-866157694) | 5.919E-13 | [System development](http://portal.genego.com/cgi/process.cgi?id=-2069873154) | 1.765E-19 |
| **5** | [Antigen processing and presentation of endogenous antigen](http://portal.genego.com/cgi/process.cgi?id=-269943210) | 1.120E-12 | [Defense response](http://portal.genego.com/cgi/process.cgi?id=-1137554385) | 1.836E-19 |
| **6** | [Immune system process](http://portal.genego.com/cgi/process.cgi?id=-966226921) | 6.894E-12 | [Response to organic substance](http://portal.genego.com/cgi/process.cgi?id=-622857804) | 1.069E-18 |
| **7** | [Regulation of protein kinase activity](http://portal.genego.com/cgi/process.cgi?id=-1349204891) | 7.220E-12 | [Single-organism developmental process](http://portal.genego.com/cgi/process.cgi?id=-1965943670) | 1.648E-18 |
| **8** | [Regulation of kinase activity](http://portal.genego.com/cgi/process.cgi?id=-816059534) | 2.077E-11 | [Multicellular organismal development](http://portal.genego.com/cgi/process.cgi?id=-528578425) | 4.361E-18 |
| **9** | [Cellular response to chemical stimulus](http://portal.genego.com/cgi/process.cgi?id=-27109587) | 2.640E-11 | [Cellular response to chemical stimulus](http://portal.genego.com/cgi/process.cgi?id=-27109587) | 5.397E-18 |
| **10** | [Defense response](http://portal.genego.com/cgi/process.cgi?id=-1137554385) | 2.666E-11 | [Regulation of multicellular organismal process](http://portal.genego.com/cgi/process.cgi?id=-140466337) | 1.262E-17 |

**Supplementary Table 3C : Pathway maps ranking**

Canonical pathway maps rank signaling and metabolic maps that are significantly in both hSOD1^G93A^ motoneurone and microglia as compared to their respective controls and significantly deregulated only in hSOD1^G93A^ motoneurone. Transcriptomic was done at symptomatic age (P90) in microglia and at the end stage of the disease (P120) for motoneurones. The top scored process has the lowest p-value.

| **Rank** | **Pathway Maps**  **(both motoneurones and microglia)** | **P value** | **Pathway Maps**  **(motoneurones only)** | **P value** |
| --- | --- | --- | --- | --- |
| **1** | [Heme metabolism](http://portal.genego.com/cgi/imagemap.cgi?id=880) | 2.368E-04 | [Immune response_Classical complement pathway](http://portal.genego.com/cgi/imagemap.cgi?id=477) | 6.852E-10 |
| **2** | [Schema: Initiation of T cell recruitment in allergic contact dermatitis](http://portal.genego.com/cgi/imagemap.cgi?id=5078) | 1.252E-03 | [Immune response_Lectin induced complement pathway](http://portal.genego.com/cgi/imagemap.cgi?id=665) | 6.123E-09 |
| **3** | [Role of ZNF202 in regulation of expression of genes involved in atherosclerosis](http://portal.genego.com/cgi/imagemap.cgi?id=703) | 1.710E-03 | [Cell cycle_Regulation of G1/S transition (part 1)](http://portal.genego.com/cgi/imagemap.cgi?id=544) | 3.565E-05 |
| **4** | [DNA damage_ATM / ATR regulation of G2 / M checkpoint](http://portal.genego.com/cgi/imagemap.cgi?id=441) | 2.622E-03 | [Immune response_Alternative complement pathway](http://portal.genego.com/cgi/imagemap.cgi?id=476) | 4.156E-05 |
| **5** | [DNA damage_Brca1 as a transcription regulator](http://portal.genego.com/cgi/imagemap.cgi?id=525) | 3.484E-03 | [Cytoskeleton remodeling_Cytoskeleton remodeling](http://portal.genego.com/cgi/imagemap.cgi?id=714) | 5.013E-05 |
| **6** | [DNA damage_ATM/ATR regulation of G1/S checkpoint](http://portal.genego.com/cgi/imagemap.cgi?id=426) | 3.958E-03 | [Cytoskeleton remodeling_Neurofilaments](http://portal.genego.com/cgi/imagemap.cgi?id=1491) | 5.024E-05 |
| **7** | [p53 signaling in Prostate Cancer](http://portal.genego.com/cgi/imagemap.cgi?id=3205) | 4.205E-03 | [Transcription_Transcription regulation of aminoacid metabolism](http://portal.genego.com/cgi/imagemap.cgi?id=470) | 5.024E-05 |
| **8** | [Immune response_Role of integrins in NK cells cytotoxicity](http://portal.genego.com/cgi/imagemap.cgi?id=2228) | 5.549E-03 | [Cytoskeleton remodeling_TGF, WNT and cytoskeletal remodeling](http://portal.genego.com/cgi/imagemap.cgi?id=715) | 9.731E-05 |
| **9** | [Transcription_P53 signaling pathway](http://portal.genego.com/cgi/imagemap.cgi?id=412) | 5.838E-03 | [Development_Transcription regulation of granulocyte development](http://portal.genego.com/cgi/imagemap.cgi?id=458) | 1.732E-04 |
| **10** | [Development_Role of Activin A in cell differentiation and proliferation](http://portal.genego.com/cgi/imagemap.cgi?id=2482) | 6.134E-03 | [IL-6 signaling in multiple myeloma](http://portal.genego.com/cgi/imagemap.cgi?id=4920) | 1.944E-04 |
