# Supplementary material for: Brca1 is expressed in human microglia and is dysregulated in human and animal model of ALS
Source: Mol Neurodegener. 2015 Aug 1;10:34. doi: 10.1186/s13024-015-0023-x (PMC4521418; doi:10.1186/s13024-015-0023-x)
Supplement: Additional file 5: Table S4. — Gene ontology enrichment and network analysis of gene dysregulation in microglia at symptomatic age (P90). In all tables the top scored categories have the lowest p-value. Table S4A: Process networks. Table S4B: Gene ontology processes and Table S4C: Pathway maps. Percentage of dysregulated genes corresponds to the ratio of dysregulated genes in our data out of annotated genes in the given category (Gene Ontology). [file 13024_2015_23_MOESM5_ESM.docx]

**Supplementary Table 4A : Process networks ranking**

Cellular and molecular processes rank pre-set network of protein interactions that are significantly deregulated at P90 in hSOD1^G93A^ microglia as compared to control microglia. The top scored process has the lowest p-value. Percentage of deregulated genes corresponds to the number of deregulated genes in our data as compared to the total number of gene in a given process network.

| **Process Networks** | **P value** | **Deregulated genes (%)** | **Genes** |
| --- | --- | --- | --- |
| [**Chemotaxis**](http://portal.genego.com/cgi/network/net_net.cgi?term=10&id=145176) | 2.099E-08 | 17 | CCL5, CCL3L1, CD43, RalA, β-catenin, MIP-1β, Gα (i)-specific peptide GPCRs, I-TAC, Integrin, PF4, CXCL14, Osteopontin, CXCL16, CCR1, G-protein α -i family, Gα(q)-specific peptide GPCRs, C3aR, PLAU (UPA), CCR2, NCAM1, IP10, IL8RB, CXCL13 |
| [**Reproduction. Feeding and neuro-hormone signaling**](http://portal.genego.com/cgi/network/net_net.cgi?term=10&id=145150) | 1.519E-05 | 11 | STAT3, Proepithelin, IGF-1, APLP2 active fragment, HIF1A, G-protein α-i1, G α (i)-specific peptide GPCRs, LPP1, Integrin, c-Myc, PKA-reg (cAMP-dependent), PAI1, G-protein α -i family, Brca1, G α (q)-specific peptide GPCRs, APLP2 precursor, CCR2, G-protein α -s, Bcl-2, LPL, BTEB1, PPAP2, CD9, TNF α |
| [**Cytoskeleton. Intermediate filaments**](http://portal.genego.com/cgi/network/net_net.cgi?term=10&id=145146) | 4.368E-05 | 16 | Plectin 1, Tubulin β, Kinesin light chain, NEFM, MICAL, Kinesin heavy chain, NEFL, Nestin, α -actinin, JNK(MAPK8-10), NEFH, α -actinin 1, Desmuslin |
| [**Cell adhesion. Leucocyte chemotaxis**](http://portal.genego.com/cgi/network/net_net.cgi?term=10&id=145115) | 8.431E-05 | 10.5 | CCL5, CCL3L1, LPA1 receptor, Tubulin β, ZAP70, MIP-1 β, G α (i)-specific peptide GPCRs, I-TAC, G α (q)-specific EDG GPCRs, α -actinin, CXCL16, CCR1, G-protein α i family, VCAM1, G α (q)-specific peptide GPCRs, CD86, Profilin, CCR2, G α (i)-specific EDG GPCRs, IP10, IL8RB, CXCL13 |
| [**Cell adhesion. Platelet endothelium leucocyte interactions**](http://portal.genego.com/cgi/network/net_net.cgi?term=10&id=145166) | 2.048E-04 | 11 | ITGAX, CCL5, CD84, PDGF-A, PDGF-C, CD34, PF4, L-selectin, Cathepsin G, P-selectin, CD72, PAI1, VCAM1, PLAU (UPA), CD68, STAT1, MMP-2, CD9, TNF- α |
| [**Transport. Iron transport**](http://portal.genego.com/cgi/network/net_net.cgi?term=10&id=145172) | 2.422E-04 | 13 | Junctin, Ferritin, STEAP4, FTL, c-Myc, NRAMP1, HIF-prolyl hydroxylase, EGLN3, FTH1, CMAH, Apotransferrin, Holotransferrin, TNF- α, Transferrin |
| [**Development. Blood vessel morphogenesis**](http://portal.genego.com/cgi/network/net_net.cgi?term=10&id=145160) | 3.933E-04 | 9.5 | STAT3, Neuropilin-2, HIF1A, β -catenin, G-protein α -i1, Jagged1, G α (i)-specific peptide GPCRs, VEGFR-1, PF4, c-Myc, CCR1, G-protein α -i family, VCAM1, HIF-prolyl hydroxylase, G α (q)-specific peptide GPCRs, EDNRB, CEACAM1, G-protein α-s, L1CAM, STAT1, G α (i)-specific EDG GPCRs, Transferrin |
| [**Development. Regulation of angiogenesis**](http://portal.genego.com/cgi/network/net_net.cgi?term=10&id=145151) | 7.339E-04 | 9.8 | p21, STAT3, PDGF-A, HIF1A, β -catenin, G-protein α -i1, G α (i)-specific peptide GPCRs, VEGFR-1, PF4, c-Myc, PAI1, CCR1, G-protein α -i family, G α (q)-specific peptide GPCRs, EDNRB, CEACAM1, Cathepsin B, Connexin 43, STAT1, IL8RB, MMP-2 |
| [**Inflammation. Jak-STAT Pathway**](http://portal.genego.com/cgi/network/net_net.cgi?term=10&id=145136) | 1.438E-03 | 9 | CCL5, CCL3L1, STAT3, IL-2R γ chain, PDGF-C, RACK1, IGF-1, Carboxypeptidase H, IL-7 receptor, CSF2RA, SOCS2, IL7RA, CCR1, PLAU (UPA), CCR2, STAT1, CSF1, TNF- α |
| [**Cell adhesion. Synaptic contact**](http://portal.genego.com/cgi/network/net_net.cgi?term=10&id=145087) | 2.829E-03 | 9 | K(+) channel, subfamily J, CNTN1 (F3), β-catenin, Formin, CASK, Profilin II, α - actinin, Presenilin 2, δ -catenin, Annexin IV, Intersectin, Profilin, Semaphorin 4B, NCAM1, L1CAM, α -actinin 1, Presenilin |

**Supplementary Table 4B: Gene ontology processes ranking**

Gene ontology (GO) processes rank cellular processes that are significantly deregulated at P90 in hSOD1^G93A^ microglia as compared to control microglia. The top scored GO process has the lowest p-value. Percentage of deregulated genes corresponds to the number of deregulated genes in our data as compared to the total number of gene in a given GO process.

| **GO Processes** | **P value** | **Deregulated genes (%)** | **Genes** |
| --- | --- | --- | --- |
| [**Immune response**](http://portal.genego.com/cgi/process.cgi?id=-195107408) | 1.260E-18 | 4 | MHC class II α chain, H2-Aa, CCL5, CCL3L1, IL-2R γ chain, CD43, H-2L(d), C1qb, Ferritin, MHC class I, GC1QBP, CD24, ZAP70, PGE2R4, Fc γ RII β, HLA-DQA1, MIP-1- β, OAS1, β -2-microglobulin, HLA-A, ENPP1, IL-7 receptor, G α (i)-specific peptide GPCRs, I-TAC, Myelin basic protein, Integrin, Tcf(Lef), PF4, ETS, Carbohydrate sulfotransferases, G α (s)-specific prostanoid GPCRs, RGS1, CLECSF9, ETS1, CXCL14, Cathepsin G, HLA-Cw3, G α (q)-specific EDG GPCRs, CCL9, IL7RA, COLEC12, CCR1, IFITM2, G α a(q)-specific peptide GPCRs, FTH1, CD86, NEDD4, CCR2, Oasl2, HLA-C, HA1B, TLR2, HLA-B, 2'-5'-oligoadenylate synthetase, G α (i)-specific EDG GPCRs, Cystatin F, PD-L1, IP10, GEM, CXCL13, TNF- α |
| [**Chemotaxis**](http://portal.genego.com/cgi/process.cgi?id=-1066349059) | 3.890E-10 | 3 | CCL5, CCL3L1, CD43, PLA2, RalA, MIP-1 β, G α (i)-specific peptide GPCRs, I-TAC, C9orf46, VEGFR-1, PF4, CXCL14, HMG1,2, CCL9, CXC L16, CCR1, G α (q)-specific peptide GPCRs, C3aR, PLAU (UPA), CCR2, L1CAM, IP10, IL8RB, CMTM6, CXCL13 |
| [**Blood coagulation**](http://portal.genego.com/cgi/process.cgi?id=-688959951) | 1.820E-09 | 7.5 | ITGAX, CD84, CD43, PDGF-A, PLA2, Kinesin light chain, Mn(III)-Apotransferrin, GC1QBP, Kinesin heavy chain, PRKAR2B, MafF, KLC1, IGF-1, TFPI, Pleckstrin, ITGA5, β -2 transferrin, G-protein α -i1, TREM1, TRIPs, Integrin, CD48, CD244, PF4, G α (s)-specific prostanoid GPCRs, Dysbindin, PKA-reg type II (cAMP-dependent), L-selectin, P-selectin, PKA-reg (cAMP-dependent), Coagulation factor XIII A, α -actinin, PAI1, G-protein α -i family, IRAG, ATP1B1, G α (q)-specific peptide GPCRs, Profilin, Apotransferrin, PLAU (UPA), Coagulation factor XIII, CD147, Holotransferrin, G-protein α -s, L1CAM, ATP1B3, α -actinin 1, CD9, KINN, Transferrin |
| [**Response to hypoxia**](http://portal.genego.com/cgi/process.cgi?id=-916626972) | 8.272E-09 | 7.45 | PDGF-A, Mn(III)-Apotransferrin, CD24, β -2 transferrin, HIF1A, ACSL6, G α (i)-specific peptide GPCRs, PPARGC1 (PGC1- α), VEGFR-1, ETS, ALDOC, ETS1, α -actinin, Presenilin 2, VCAM1, HIF-prolyl hydroxylase, ATP1B1, EGLN3, G α (q)-specific peptide GPCRs, Apotransferrin, PLAU (UPA), Acyl-CoA synthetase, CCR2, Holotransferrin, Bcl-2, TLR2, MeCP2, MMP-2, Presenilin, TNF- α, Transferrin |
| [**Innate immune response**](http://portal.genego.com/cgi/process.cgi?id=-866848204) | 8.746E-09 | 5.8 | p21, TNRC6B, LLIR, Mx1, PDGF-A, C1qb, PLA2, BST2, MHC class I, Myosin I, DAF, GC1QBP, PRKAR2B, Dectin-1, β -catenin, OAS1, β -2-microglobulin, MIF, G α (i)-specific peptide GPCRs, TREM1, Integrin, UFO, PKA-reg type II (cAMP-dependent), CD94, HLA-Cw3, S100B, PKA-reg (cAMP-dependent), HMG1,2, SIRPB1, JNK(MAPK8-10), COLEC12, IFITM2, ISG54, CLEC10A, IIGP, RSAD2, NIPK, Adenylate cyclase, G α (q)-specific peptide GPCRs, CD86, JNK3(MAPK10), CCR2, Oasl2, HLA-C, MASP1, Cathepsin B, IFIT1, Bcl-2, TLR2, HLA-B, 2'-5'-oligoadenylate synthetase, RIG-G, Adenylate cyclase type III, CSF1, OST48, TREM2, ARP3 |
| [**Cell adhesion**](http://portal.genego.com/cgi/process.cgi?id=-552545827) | 1.035E-08 | 5.49 | ITGAX, CD84, LLIR, 90K, Neuropilin-2, OSF-2, CD229, CD24, CNTN1 (F3), ITGA5, β -catenin, MIP-1 β, β -IG-H3, CASK, G α (i)-specific peptide GPCRs, CD22, Integrin, CD34, Tcf(Lef), Carbohydrate sulfotransferases, Migfilin, L-selectin, BMX, Osteopontin, P-selectin, Flotillin-2, CD72, α -actinin, CCR1, VCAM1, TSLC1, δ-catenin, ATP1B1, MIC2, G α (q)-specific peptide GPCRs, LAMA3 (Epiligrin), CEACAM1, FN14(TNFRSF12A), C1qRp, NCAM1, CASS4, L1CAM, CAS-L, KLRA1, G α (i)-specific EDG GPCRs, PPAP2, Nectin-3, Presenilin, CD36L2, CD9, RhoE, CD166, S2V, NPNT |
| [**Iron ion transport**](http://portal.genego.com/cgi/process.cgi?id=-1651018785) | 1.286E-08 | 13 | Ferritin, Mn(III)-Apotransferrin, β -2 transferrin, FTL, Ferritin light chain (MOUSE), NRAMP1, FTH1, Apotransferrin, Holotransferrin, Transferrin |
| [**Response to IFN-γ**](http://portal.genego.com/cgi/process.cgi?id=-1940603868) | 1.737E-08 | 6.7 | MHC class II α chain, H2-Aa, CCL5, BST2, HLA-DQA1, α -synuclein, HMG1,2, NRAMP1, CXCL16, IFITM2, CD86 |
| [**Ferric iron transport**](http://portal.genego.com/cgi/process.cgi?id=-1943411187) | 2.792E-08 | 10.6 | Mn(III)-Apotransferrin, β -2 transferrin, Apotransferrin, Holotransferrin, Transferrin |
| [**Platelet degranulation**](http://portal.genego.com/cgi/process.cgi?id=-1657266127) | 3.068E-08 | 15.1 | PDGF-A, Mn(III)-Apotransferrin, IGF-1, Pleckstrin, β -2 transferrin, PF4, P-selectin, Coagulation factor XIII A, α -actinin, PAI1, Profilin, Apotransferrin, Coagulation factor XIII, |

**Supplementary Table 4C: Pathway maps ranking**

Canonical pathway maps rank signaling and metabolic maps that are significantly deregulated at P90 in hSOD1^G93A^ microglia as compared to control microglia. The top scored map has the lowest p-value. Percentage of deregulated genes corresponds to the number of deregulated genes in our data as compared to the total number of gene in a given pathway map.

| **Pathway Maps** | **P value** | **Deregulated genes (%)** | **Genes** |
| --- | --- | --- | --- |
| **Cytoskeleton remodeling,**  **Neurofilaments** | 2.657E-07 | 32 | PGE2R4, HIF1A, β -catenin, Tcf(Lef), c-Myc, PKA-reg (cAMP-dependent), G-protein α -i family, Adenylate cyclase, TCF7L2 (TCF4), G-protein α -s, TNF- α |
| [**PGE2 pathways in cancer**](http://portal.genego.com/cgi/imagemap.cgi?id=2373) | 3.023E-07 | 20 | PGE2R4, HIF1A, β -catenin, Tcf(Lef), c-Myc, PKA-reg (cAMP-dependent), G-protein α -i family, Adenylate cyclase, TCF7L2 (TCF4), G-protein α -s, TNF- α |
| [**DNA damage_Brca1 as a transcription regulator**](http://portal.genego.com/cgi/imagemap.cgi?id=525) | 1.577E-05 | 23 | p21, PCNA, GADD45 α, c-Myc, Brca1, STAT1, SP3 |
| [**Cell adhesion. Chemokines and adhesion**](http://portal.genego.com/cgi/imagemap.cgi?id=716) | 2.372E-05 | 12 | β -catenin, Tcf(Lef), c-Myc, Flotillin-2, α -actinin, PAI1, CCR1, G-protein α -i family, PLAU (UPA), α -actinin 1, IL8RB, MMP-2 |
| [**Development_G-CSF signaling**](http://portal.genego.com/cgi/imagemap.cgi?id=6413) | 6.147E-05 | 16 | STAT3, BFL1, c-Myc, JNK(MAPK8-10), Bcl-2, STAT1, GFI-1, IL8RB |
| [**Immune response_Histamine signaling in dendritic cells**](http://portal.genego.com/cgi/imagemap.cgi?id=2438) | 7.142E-05 | 16 | CCL5, MIP-1β, PKA-reg (cAMP-dependent), G-protein α -i family, Adenylate cyclase, CD86, G-protein α -s, TNF- α |
| [**Transcription_P53 signaling pathway**](http://portal.genego.com/cgi/imagemap.cgi?id=412) | 9.580E-05 | 18 | p21, β-catenin, VEGFR-1, JNK(MAPK8-10), MAP4, Bcl-2, MMP-2 |
| [**DNA damage_ATM/ATR regulation of G1/S checkpoint**](http://portal.genego.com/cgi/imagemap.cgi?id=426) | 2.385E-04 | 18.7 | p21, CDK4, PCNA, GADD45 α, c-Myc, Brca1 |
| **Role of ZNF202 in regulation of expression of genes involved in atherosclerosis** | 2.469E-04 | 24 | ABCG1, APOE, LPL, HDL proteins, PLTP |
| **Immune response_IL-22 signaling pathway** | 3.376E-04 | 17.6 | STAT3, c-Myc, JNK(MAPK8-10), CD86, Bcl-2, STAT1 |
