# Supplementary material for: Brca1 is expressed in human microglia and is dysregulated in human and animal model of ALS
Source: Mol Neurodegener. 2015 Aug 1;10:34. doi: 10.1186/s13024-015-0023-x (PMC4521418; doi:10.1186/s13024-015-0023-x)
Supplement: Additional file 7: Table S5. — Candidate genes involved in Brca1 pathway selected for qPCR to validate the microarray data. [file 13024_2015_23_MOESM7_ESM.docx]

**Supplementary Table 5 -** Candidate genes involved in Brca1 pathway selected for qPCR to validate the microarray data.

| **Gene symbol** | **Description** | **Primer sequences**  **(**F: Forward (5’-3’), R: Reverse(5’-3’)) |
| --- | --- | --- |
| *Brca1* | Breast cancer 1 | F: ATCAGTGCTCCTCAAGGCTCC  R : GGGTGTTGTTTGGTGCTGG |
| *Cdkn1a* | Cyclin-dependent kinase inhibitor 1A (P21) | F: ACGTGGCCTTGTCGCTGT  R: AATCTTCAGGCCGCTCAGAC |
| *Myc* | Myelocytomatosis oncogene | F: GAGGCGGACACACAACGTCT  R: CGCTTCAGCTCGTTCCTCC |
| *Pcna* | proliferating cell nuclear antigen | F: TAAAGATGCCGTCGGGTGA  R: TGTGGCTAAGGTCTCGGCATA |
| *Stat1* | Signal transducer and activator of transcription 1 | F: GTGCCTCTGGAATGATGGGT  R: TCTCGCTCCTTGCTGATGAAG |
| *Gadd45a* | Growth arrest and DNA-damage-inducible 45 α | F: GTTCAACTGCACGAGGGCTC  R: CACCTCTCTCTCCCTCTGCAAA |
| *Sp3* | Trans-acting transcription factor 3 | F:TTGGGTGGTAAAATGTTCTAGTGAAT  R: CCTCCAAACTTCAGGAAAAGGG |
| **Housekeeping genes** | | |
| *TBP* | TATA box binding protein | F: TTGACCTAAAGACCATTGCACTTC  R: TTCTCATGATGACTGCAGCAAA |
| *RPS9* | Mitochondrial ribosomal protein S9 | F: GACCAGGAGCTAAAGTTGATTGGA  R: TCTTGGCCAGGGTAAACTTGA |
| *Actin β* | Actin b | F: GACCAGGAGCTAAAGTTGATTGGA  R: CACAGCCTGGATGGCTACGT |
| *EEF1* | Eukaryotic translation elongation factor 1 | F: TCCACTTGGTCGCTTTGCT  R: CTTCTTGTCCACAGCTTTGATGA |
